# Video as a public health knowledge transfer tool in Burkina Faso: A mixed evaluation comparing three narrative genres

**DOI:** 10.1371/journal.pntd.0008305

**Published:** 2020-06-10

**Authors:** Catherine Hébert, Christian Dagenais, Esther Mc Sween-Cadieux, Valéry Ridde

**Affiliations:** 1 Department of Psychology, University of Montreal, Montreal, Quebec, Canada; 2 French Institute For Research on sustainable Development (IRD), CEPED (IRD-Université Paris Descartes), Universités Paris Sorbonne Cités, ERL INSERM SAGESUD, Paris, France; National Center for Atmospheric Research, UNITED STATES

## Abstract

**Background:**

The dengue virus is endemic in many low- and middle-income countries. In Burkina Faso, the proportion of fevers that could be due to dengue is growing. In 2013, a dengue epidemic spread there, followed by other seasonal outbreaks. Dengue is often confused with malaria, and health workers are not trained to distinguish between them. Three training videos using different narrative genres were tested with nursing students from two institutions in Ouagadougou: journalistic, dramatic and animated video. The study aimed to determine if video is an effective knowledge transfer tool, if narrative genre plays a role in knowledge acquisition, and which narrative elements are the most appreciated.

**Methodology:**

A mixed method research design was used. The relative effectiveness of the videos was verified through a quasi-experimental quantitative component with a comparison group and post-test measurements. A qualitative component identified participants’ perceptions regarding the three videos. Data were drawn from a knowledge test (n = 482), three focus groups with health professionals’ students (n = 46), and individual interviews with health professionals (n = 10). Descriptive statistics and single-factor variance analysis were produced. A thematic analysis was used to analyse qualitative data.

**Principal findings:**

Results showed that all three videos led to significant rates of knowledge improvement when compared with the comparison group (*p* <0.05): 12.31% for the journalistic video, 20.58% for the dramatic video, and 18.91% for the animated video. The dramatic and animated videos produced a significantly higher increase in knowledge than did the journalistic video (with respectively 8.27% (*p* = 0.003) and 6.59% (*p* = 0.029) and can be considered equivalent with a difference of 1.68% (*p* = 0.895). Thematic analysis also revealed that these two videos were considered to be better knowledge transfer tools. Four key aspects are important to consider for a video to be effective: 1) transmitting information in a narrative form, 2) choosing good communicators, 3) creating a visual instrument that reinforces the message and 4) adapting the message to the local context.

**Conclusions:**

Video has proven to be an effective and appreciated knowledge transfer and training tool for health professionals, but the narrative genre of the videos can influence knowledge acquisition. The production of other videos should be considered for training or updating health professionals and their narrative genre taken into consideration. The actual context of constant circulation of new diseases, such as COVID-19, reaffirms the need to train health professionals.

## Author summary

The dengue virus is endemic in many low- and middle-income countries, including Burkina Faso. Since 2013, successive seasonal epidemics in the country have resulted in many deaths that could have been prevented if health professionals had been better trained. Although recent studies have reported the presence of the virus in Burkina Faso's capital, Ouagadougou, several cases of dengue fever have been reported that were misdiagnosed and treated incorrectly. Video is a promising knowledge transfer (KT) tool to address the training needs of health workers. The ease with which a video can be shared, and its playful nature makes it a particularly attractive tool for reaching and training them and transforming their practices, especially, given their busy schedules. The study addresses the use of video as a KT strategy in relation to the narrative genre, i.e., how information is presented. It contributes to the advancement of knowledge on what motivates the use of video as a KT tool and practice changes in working contexts where professionals need to know how to respond, quickly and appropriately, to health problems such as dengue and other emerging pathologies in Africa.

## Introduction

Transferring research evidence into practice is often a slow and complex process. Despite numerous efforts over recent decades, there remains a substantial gap between research evidence production and use [1, 2]. When this gap widens, consequences can be dramatic. In the health field, for example, patients may be deprived of treatments that have been proven effective [3]. A viral disease such as dengue fever, particularly prevalent in Burkina Faso, can be fatal if misdiagnosed. Dengue fatalities are, however, considered to be avoidable in 99% of cases [4]. In 2014, researchers concluded that Burkinabè health workers lacked training on dengue fever and that interventions were urgently needed to transfer existing knowledge to them [5].

The question, however, is how best to do this. It has already been established that researchers concerned with knowledge transfer (KT) need to develop strategies to promote knowledge uptake [6, 7], including audiovisual documents (videos). While the effectiveness of video as a KT tool in public health has already been demonstrated [8], there has been little research on what narrative genre best supports knowledge transmission and retention: journalistic, dramatic, or animated infographics. How best to design information and present data visually to bridge the gap between scientists and their audiences remains a core question [9]. As such, it is relevant to study what narrative genre is most useful to make videos an effective KT tool geared specifically to health workers in Burkina Faso.

### Global incidence of dengue

Dengue is a mosquito-borne viral infection that has dramatically spread around the world in recent decades, primarily in the tropics and subtropics [10, 12]. Even if the epidemiological patterns of this disease are alarming, its global burden is still not well known [13, 14]. According to one model, there are an estimated of 390 million dengue virus infections globally per year (95% credible interval 284–528 million). Of these, 96 million (67–136 million) (approximately 25%) will have clinical signs and symptoms of the disease (e.g., fever, severe headache, nausea, muscle and joint pain, rash, pain behind the eyes) [10, 14]. Around one in ten cases will progress to a more severe form of the disease such as dengue hemorrhagic fever and dengue shock syndrome [14–16]. Each year, it is estimated that 10,000–20,000 people die from dengue in the world [10, 11, 13]. There is still no effective antiviral treatment for dengue fever [12] and the protection offered by the licensed vaccine is incomplete and is serotype-specific [17]. Dengue transmission can be controlled by vectors surveillance and monitoring, and the fatality rates can be lowered below 1% with an appropriate medical management of the diseases [12].

The majority of cases are asymptomatic or mild and self-managed and the mortality rate from severe dengue is relatively low. Yet, the economic and resource burden on health services are important in many endemic settings [10, 18]. Moreover, around half the world’s population would be at risk of infection with dengue viruses annually (3.9 billion) [10, 11, 18]. This risk of infection is present in 129 countries, but 70% of the current burden is in the Asian region [10]. However, certain modelling frameworks suggest that the disease burden could be similar in Africa, but without good surveillance systems, the situation is still unknown in this region [14, 17, 19]. Although dengue is considered a neglected tropical disease, it requires more attention from policymakers, researchers and health workers especially in low- and middle-income countries. There is a need to strengthen the infrastructure for surveillance, case reporting and diagnosis [12, 14, 19]. It is highly probable that dengue cases are underreported, and many cases are misdiagnosed [10, 20] due to the presence of other febrile illnesses.

### Dengue in Burkina Faso (West Africa)

In Burkina Faso, dengue and other hemorrhagic fevers’ surveillance faces multiple obstacles such as a general lack of knowledge about dengue in the health system, lack of diagnostic means and the limited health workers’ training [21, 22]. Available data seem to show a significant morbidity and mortality in the country [23]. Following the dengue epidemic in 2013 [24], a new outbreak was reported in 2016 [25] and in 2017. According to the World Health Organization, 1266 suspected cases, 1061 positive cases by dengue rapid diagnostic test (RDT: TDR in French) and 15 deaths were reported in 2016 [26]. In 2017, 9 029 suspected cases, 5773 dengue RDT-positive cases and 18 deaths (case fatality rate = 0.2%) were reported throughout the country [26, 27]. The number of deaths due to dengue would likely have been lower if health workers had been better trained. Dengue fever is often confused with malaria, as the symptoms of the two diseases are similar, but taking antimalarial drugs can worsen the condition of a patient with dengue fever [28]. Unfortunately, even though recent studies have reported the presence of the dengue virus in Ouagadougou, the capital of Burkina Faso [29, 30], many cases of dengue continue to be misdiagnosed and improperly treated [22, 31]. Very few health workers have received training on dengue fever or non-malaria febrile diseases [21, 22]. Thus, healthcare providers still know very little about dengue fever.

### Video as a knowledge transfer tool

For decades, research has shown that video can improve people's knowledge on health topics, can promote behavior change, can increase self-efficacy and many other advantages [32]. This can be explained by the fact that audiovisual materials are able to convey complex arguments and stimulate new reflections [33]. Based on brain activity analyses and cognitive tests, a neurocognition research [34] demonstrated that, when viewing a video, learners develop a cognitive activity that reinforces memorization and problem-solving processes. Moreover, research has confirmed that viewing video improves the audience’s ability to visualize a phenomenon and commit different learning experiences to memory [35]. A recent meta-analysis on health communication suggests that narratives delivered via audio and video would be more persuasive than a print information [36].

Furthermore, interventions via mobile phones are increasingly used in health promotion campaigns because they offer the possibility of disseminating automated, timely and specific messages to a target audience [37]. Moreover, Mobile health (*Mhealth*) has gained more and more attention as an innovative technology to promote health by supporting healthcare practices [38]. The use of mobile video delivery is also promising because this tool is less expensive than traditional training methods—such as a face-to-face presentation—especially when a significant number of people need to be trained [39]. Video as a KT tool also has the advantage of being easy to distribute in a context where videos are widely shared via mobile phones and social media [40]. Of course, for a video to be effective, it must circulate and be viewed. Over the past decade, the use of mobile phones to support public health practices has become increasingly common in low-income countries, particularly in Africa [41, 42]. In Burkina Faso, nearly 95 out of 100 inhabitants are mobile telephone service subscribers in 2019 [43].

As access to mobile phones is still improving in resource-constrained settings, more research is needed to better understand the potential of these promising technologies to improve healthcare systems [40], and to address the training needs of health workers. On that point, some studies in Africa have measured the effectiveness of video in improving farmers' knowledge [39], [44]. These studies concluded that video is an effective KT tool and could be used to supplement or replace traditional training. In summary, although the use of video is widespread in Africa with the growing popularity of social media, and its effectiveness has often been studied in comparison to traditional training (such as lectures), we have not identified studies that precisely assess the effectiveness of this KT tool based on its narrative genre, i.e., how information is presented.

### Objectives and research questions

The objective of this article is to present findings regarding the effectiveness of three videos by responding to the following research questions: 1) Is video an effective KT tool for transmitting research evidence on dengue to health workers in Burkina Faso? 2) Did the participants’ learning vary depending on which video they viewed? 3) What narrative elements (e.g., narration, images, infographics) make a video a more effective KT tool?

## Methods

### Design

The study was conducted using a mixed methodology [45] with multiple data sources to increase the validity and quality of the results [46] and to develop more in-depth knowledge of the existing situation [47]. The relative effectiveness of each video was verified through the quasi-experimental quantitative component with a comparison group (post-test only control-group design). The qualitative component identified participants’ perceptions regarding each video. A convergent design was adopted that combined qualitative and quantitative methods from the outset of the planning phase of the study [48]. Quantitative and qualitative data collections were done separately, but the results were combined for analysis and interpretation.

### Development and description of the videos

The three videos were designed in several stages. First, to develop the content of the videos, we reviewed literature on dengue fever. A narrative text was then drafted and submitted to a committee of four experts working on dengue fever from Canada and Burkina Faso. The time required to read this text, which contained information considered essential by experts on the transmission and treatment of the virus, was estimated as four and a half minutes. A scenario was prepared for each video, all using the same text.

**Fig 1:**
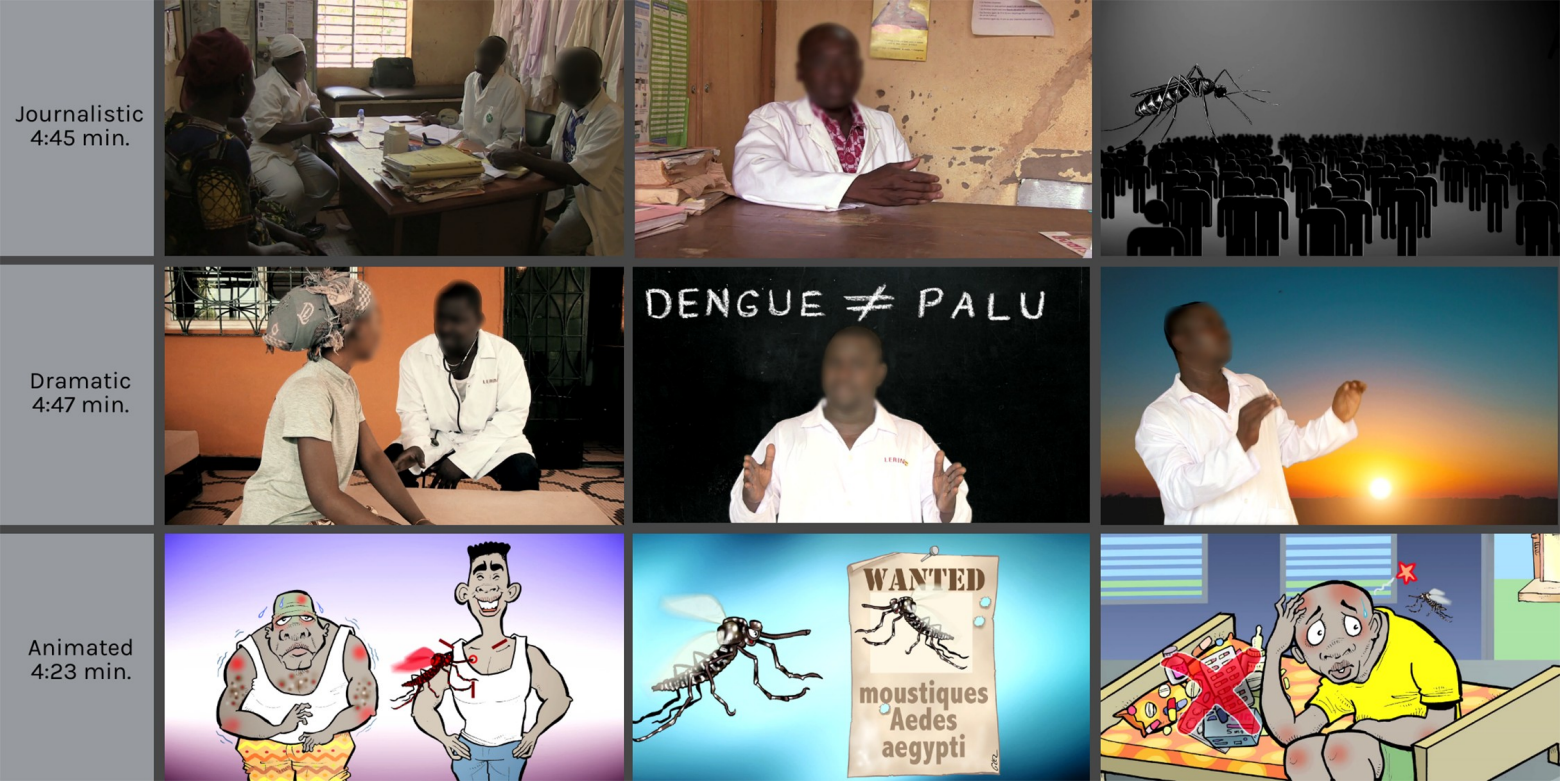
Overview of the narrative genres of the videos.

Thus, the three videos conveyed the same scientific information, but each used a different narrative genre (Fig 1). Video 1 took the form of a journalistic report. Video 2 was a dramatization in which two Burkinabè actors enacted a conversation between a physician and a patient presenting with dengue fever symptoms. Video 3 was an animated film using comic illustrations. These narrative genres were selected not only because they allowed us to compare three very distinct visual and narrative options, but also because they corresponded to two basic media genres: information (journalism) and fiction (dramatization, animation) [49].

The videos were recorded in Ouagadougou in October 2016. Video 1 (journalistic) was narrated by a female Burkinabè voice and showed generic images recorded in a health centre (waiting room, consultation, rapid screening test, nurses, pharmacy), in an interior courtyard (tire, water canister, plant pot), and at the market (crowd, fruit and vegetable vendors). This video also featured a nurse being interviewed and included two short infographic elements. In video 2 (dramatic), the consultation scenes alternated with monologues by the physician directly addressing the camera. The script was edited with the actor who played the physician, and the video was recorded in a private home. The physician’s monologues were filmed against a green screen to allow images to be inserted into the background at the postproduction stage. Video 3 (animated) used characters created by the French-Burkinabè cartoonist Damien Glez. It was narrated by a European male voice. The drawings went through several iterations before the final versions were approved by the research team. In Montreal, a professional animator (motion designer) animated these characters using *Adobe After Effects* software and produced a soundtrack with special effects.

### Measurement instruments

For the quantitative component, a questionnaire (see Appendix 1) was developed to test participants’ knowledge. It consisted of 21 questions: six socio-demographic, 10 multiple-choice (a, b, c, d), and five true-or-false questions. The questions were developed in order to obtain a knowledge score and covered the information provided in the videos (such as the nesting sites of the mosquito carrying the dengue virus). The questionnaire was developed with three dengue experts in Ouagadougou and Montreal. It was pretested with five African students from the Université de Montréal, and minor modifications were made to the wording of certain questions to adapt them to the Burkinabè context.

A discussion grid (see Appendix 2) was developed for the qualitative component. It focused on the participants’ assessment of the videos and the different narrative elements that constituted them: speakers and narration, visual aspects and graphic elements (real-life images, infographics, animations), language, and tone used (serious, humorous).

### Sampling of participants–quantitative component

**Fig 2:**
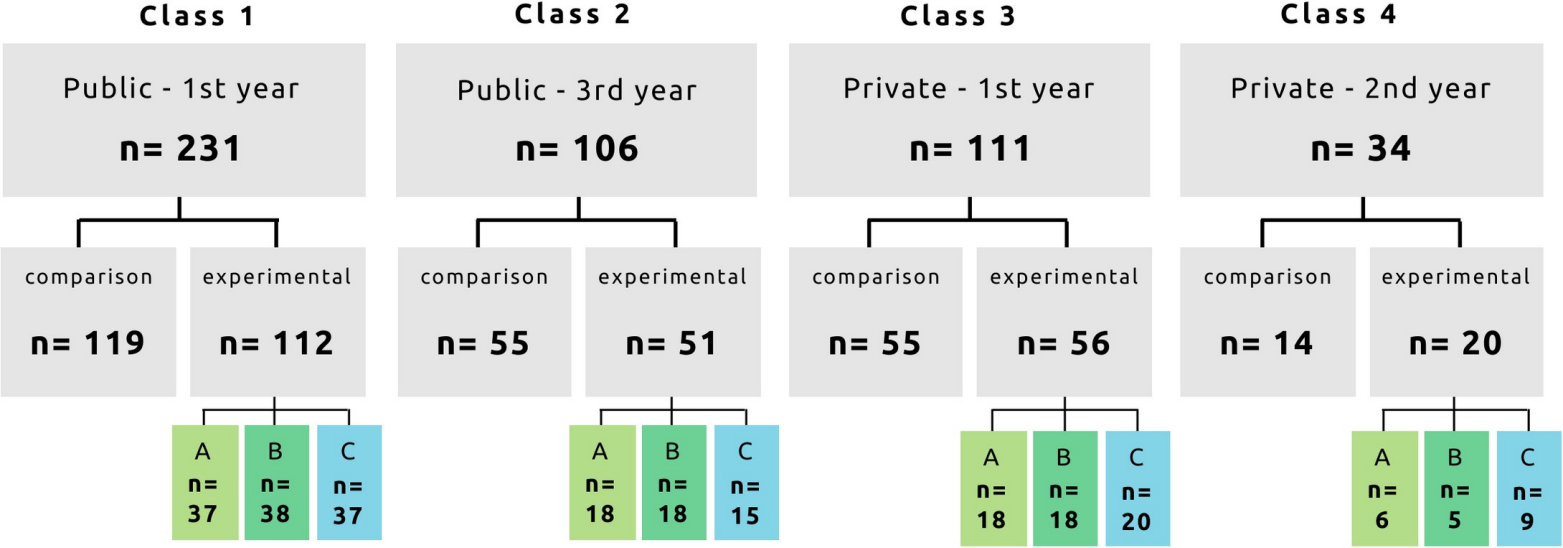
Distribution of study participants for the quantitative component (n = 482). Group A–video 1 journalistic; Group B–video 2 dramatic; Group C–video 3 animation.

The research was conducted with nursing students from two institutions in Ouagadougou: the *École Nationale de Santé Publique* and the *École Privée de Santé Sainte-Julie*. The former school was chosen because it is the largest public institution for the training of non-physician health personnel. The latter one, a private school, was added to enlarge and diversify the sample. The study was conducted using a non-randomized convenience sample to respect the distribution into groups (Fig 2). The videos were screened in rooms at the university with four groups of students enrolled in the nursing program: two classes at the public school (1^st^ and 3^rd^ years) and two classes at the private school (1^st^ and 2^nd^ years), for a total of 482 participants.

First-year students made up the majority of the sample (*n* = 342, 71%). More than two-thirds of the sample came from the public school (*n* = 337, 70%), and most were female (n = 335, 71%). Participants were generally between 20 and 30 years of age (*n* = 409, 85%). Two-thirds of them (*n* = 297, 64%) had no urban experience, and three-quarters (*n* = 361, 76%) had no rural experience. Most (*n* = 429, 89%) had completed a secondary education (BEPC or Baccalauréat) (Table 1).

**Table 1:** Sample characteristics for the quantitative component (n = 482).

		No video n = 243	Journalistic n = 79	Dramatic n = 79	Animated n = 81	Total n = 482
**University year** (n = 482)	1^st^ year	174 (71.6%)	55 (69.6%)	56 (70.9%)	57 (70.4%)	342
	2^nd^ year	14 (5.8%)	6 (7.6%)	5 (6.3%)	9 (11.1%)	34
	3^rd^ year	55 (22.6%)	18 (22.8%)	18 (22.8%)	15 (18.5%)	106
**Institution type** (n = 481)	Public	175 (72.0%)	55 (69.6%)	55 (70.5%)	52 (64.2%)	337
	Private	68 (28.0%)	24 (30.4%)	23 (29.5%)	29 (35.8%)	144
**Sex** (n = 480)	Female	165 (68.2%)	50 (64.1%)	58 (73.4%)	62 (76.5%)	335
	Male	77 (31.8%)	28 (35.9%)	21 (26.6%)	19 (23.5%)	145
**Age** (n = 480)	21–25 years	114 (47.1%)	43 (54.4%)	39 (50.0%)	47 (58.0%)	243
	26–30 years	91 (37.6%)	20 (25.3%)	30 (38.5%)	25 (30.9%)	166
	31–35 years	32 (13.2%)	12 (15.2%)	9 (11.5%)	7 (8.6%)	60
	>35 years	5 (2.1%)	4 (5.1%)	0 (0.0%)	2 (2.5%)	11
**Years of work experience in urban areas** (n = 472)	0	145 (61.4%)	47 (59.5%)	53 (67.9%)	52 (65.8%)	297
	1–2	11 (4.7%)	7 (8.9%)	5 (6.4%)	4 (5.1%)	27
	3–4	20 (8.5%)	11 (13.9%)	6 (7.7%)	1 (1.3%)	38
	5–6	8 (3.4%)	3 (3.8%)	2 (2.6%)	4 (5.1%)	17
	>6	52 (22.0%)	11 (13.9%)	12 (15.4%)	18 (22.8%)	93
**Years of work experience in rural areas** (n = 475)	0	181 (76.1%)	54 (69.2%)	65 (82.3%)	61 (76.3%)	361
	1–2	10 (4.2%)	6 (7.7%)	4 (5.1%)	7 (8.8%)	27
	3–4	15 (6.3%)	5 (6.4%)	3 (3.8%)	5 (6.3%)	28
	5–6	6 (2.5%)	4 (5.1%)	2 (2.5%)	1 (1.3%)	13
	>6	26 (10.9%)	9 (11.5%)	5 (6.3%)	6 (7.5%)	46
**Highest diploma obtained** (n = 477)	Primary—CEP	0 (0.0%)	1 (1.3%)	0 (0.0%)	0 (0.0%)	1
	Secondary 1 –BEPC (CAP, BEP)	79 (32.8%)	16 (20.8%)	25 (31.6%)	26 (32.5%)	146
	Secondary 2—Baccalauréat	143 (59.3%)	47 (61.0%)	45 (57.0%)	48 (60.0%)	283
	DEUG/Licence	15 (6.2%)	11 (14.3%)	9 (11.4%)	5 (6.3%)	40
	Master 1/DESS-DEA (Master 2)	4 (1.7%)	2 (2.6%)	0 (0.0%)	1 (1.3%)	7

* Note that the percentages in brackets are column percentages

**Abbreviations: Primary school** (CEP–Certificat d’études primaires)**; Secondary 1** (BEPC–Brevet d’études du premier cycle; CAP- Certificat d’aptitudes professionnel; BEP–Brevet d’études professionnel)**; Secondary 2** (Baccalauréat); **Post-secondary/higher education (**undergraduate: DEUG–Diplôme d’études universitaires générales, graduate: DESS–Diplôme d’études supérieures spécialisées, DEA- Diplôme d’études approfondies)

### Procedure

#### Quantitative component

**Fig 3:**
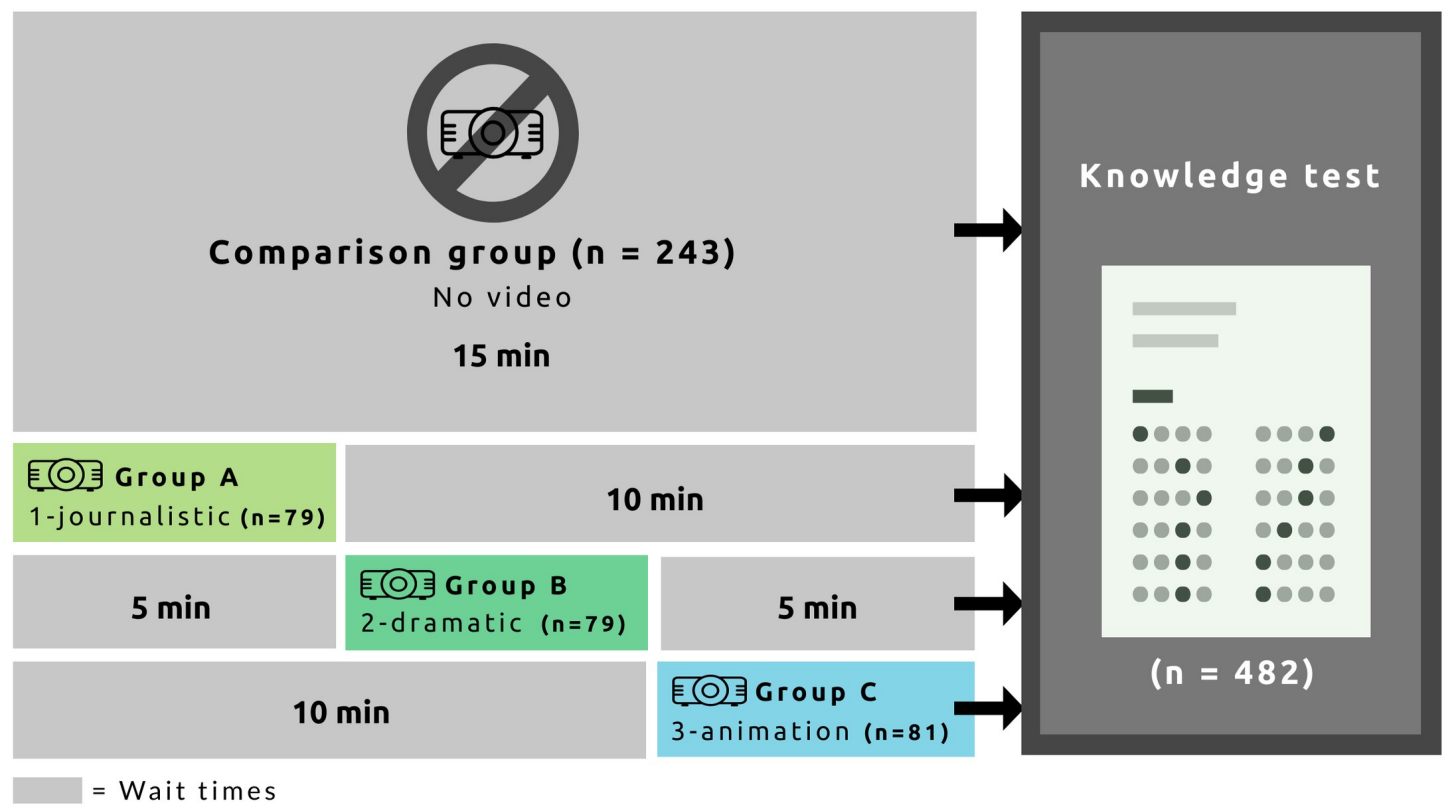
Procedure for participants’ wait times for the quantitative component.

In a first step, the students from each class were divided into two groups: comparison and experimental, with the latter further divided into three subgroups (Fig 2). For purposes of efficiency and to minimize wait times, the procedure was tested with the first class (public school - 3rd year) and then reviewed and revised. In that initial round, the comparison group was first brought into the classroom to complete the knowledge test without having seen any video, after which they left the room. Then, in sequence, each experimental group entered the room to view one video: Group A, video 1; Group B, video 2; Group C, video 3. The order of presentation of the videos was modified for each of the three groups (i.e., in the second group, Group A watched video 2, Group B watched video 3, etc.). Each group completed the knowledge test immediately after viewing their video and then vacated the room for the next group. After this trial run, the timing was modified for the remaining three classes. In these rounds, the three experimental groups each viewed the video assigned to them, without completing the knowledge test immediately afterward (Fig 3). After all three screenings had been completed, all the students—including the comparison group—were invited into the room to complete the knowledge test. Two versions of the test were distributed to the participants to ensure the integrity of the results. Distribution of the two versions was alternated to avoid the possibility of plagiarism.

#### Qualitative component

Three focus groups were formed on a voluntary basis (Table 2), made up of Burkinabè university students between the ages of 20 and 25 years (n = 46). Only those who had not seen any video (comparison group) were recruited. In these groups, all three videos were screened, followed by a discussion lasting 45 to 75 minutes. Participants were first asked to indicate which video they preferred. The facilitator began by inviting those who had preferred the least popular video to speak, so they could express their opinions without being influenced by the rest of the group. This was followed by a group discussion based on the discussion grid presented above (see Appendix 2). Ten semi-structured interviews were also conducted (45–60 minutes each), using the same grid, with four instructors and six nurse volunteers (Table 3). These respondents were carefully recruited by the local research coordinator in Burkina Faso based on certain criteria such as being in contact with patients (possibly suffering from dengue fever) or knowing the health professionals' training.

**Table 2:** Composition of the three focus groups (n = 46).

Public school– 1st year class	21 participants (8 female, 13 male)
Public school– 3rd year class	14 participants (6 female, 8 male)
Private school– 2nd year class	11 participants (4 female, 7 male)

**Table 3:** Description of persons interviewed (n = 10).

Instructor (2nd and 3rd year)	Public school
Instructor, Head of training for State-certified nurses	Public school
Instructor (3rd year)	Public and private schools
Nurse manager	Hospital
Coordinator of obstetric nursing	Public school
Nurse	Primary health centre
Head nurse	Primary health centre
Nurse	Primary health centre
Nurse	Primary health centre
Nurse	Primary health centre

### Data analysis

The repartition of participants into groups was not done at random, but groups are not statistically different for all characteristics in Table 1 (all chi square tests were non-significant; *p* > 0.192). For each group (no video, journalistic, dramatic, animated), to assess knowledge acquisition we compared the total correct responses for each student. We then carried out the same means analyses for each question. To do this, we performed a single-factor intersubject variance analysis (no video, journalistic, dramatic, animated), followed by Tukey’s post hoc testing. Lastly, we investigated the effects of interactions between variables. The analyses were performed with SPSS version 24 software using a 5% alpha significance threshold.

To answer the third research question (qualitative component), the focus group sessions and individual interviews were digitally recorded. These audio files were imported into *NVivo 10* qualitative data processing software and then partially transcribed by the first author who also conducted the interviews. The statements collected were analyzed using a thematic analysis method. Based on meticulous reading of the contents, all themes raised in the discussions were identified, grouped, and structured [50]. Each theme was summarized to better understand the different names given to things, the participants’ reasoning, and the factors that influenced their perceptions. The analysis of the partial transcript of a focus group was submitted to a research professional and was the subject of an interjurisdictional agreement.

The quantitative and qualitative results were analyzed using a triangulation approach [51]. Applying a results comparison strategy [48], we compared the similarities and differences in the results of the two components. The quantitative and qualitative results were analyzed in parallel to study the same object, before being combined at the interpretation stage to enhance the depth of the conclusions. For example, qualitative results were useful to better understand or explain certain quantitative results (for example, the least successful questions, the preference for a certain narrative genre, etc.).

Ethics certificates were obtained from the research ethics committees of the University of Montreal Hospital Research Centre (15.190) and the National Health Ethics Committee of Burkina Faso (2015-10-06). Informed consent to participate was obtained from participants.

## Results

The results of our study are presented in two sections. The first, focusing on the quantitative component, reports on the videos that led to the highest scores on the knowledge test. Then the qualitative component is presented in terms of the themes raised; in that section, certain statistical results are provided to support our analysis.

### Quantitative component

For questions 1 to 10 (multiple choice), statistically significant differences were seen among the four groups (F(3, 478) > 4.614; *p* <0.003). For questions 11 to 15 (true-or-false), only question 12 showed a statistically significant difference among the groups (F(3, 478) = 2.986, *p* = .031). Questions 11, 13, and 14 showed no statistically significant difference among the groups (F(3, 478) < 2.155, *p* >.092). For question 15, data were missing, as several participants did not respond. No statistically significant differences were seen among the groups for this question (F(3, 288) = 0.630, *p* = .596). For participants who did not respond to question 15, the result of their average for the 14 first questions was used (Table 4). This is the only question from the knowledge test with missing data.

**Table 4:** Results obtained by each group at the knowledge test–Mean % (standard deviation) [95% confidence interval].

	No video (n = 243)	Journalistic (n = 79)	Dramatic (n = 79)	Animated (n = 81)
**Q1**—The risk of being infected with dengue fever is highest *(at night/at sunrise/at sunset/at sunrise and sunset)*	**25.51** (43.84) a [19.99 ;31.03]	**43.04** (49.83) b [31.88 ;54.20]	**65.82** (47.73) c [55.13 ;76.51]	**41.98** (49.66) b [30.99 ;52.96]
**Q2—**The mosquito responsible for dengue fever transmission lays its eggs *(rain puddles/ponds/****everyday containers****/large bodies of water/all)*	**24.69** (43.21) a [19.23 ;30.15]	**24.05** (43.01) a [14.42 ;33.68]	**37.97** (48.84) a,b [27.03 ;48.91]	**44.44** (50) b [33.39 ;55.50]
**Q3—**Every year, globally, the number of deaths due to dengue fever is estimated to be *(10 000/****20 000****/30 000/more than 30 000)*	**25.93** (43.91) a [20.38 ;31.47]	**67.09** (47.29) b [56.50 ;77.68]	**50.63** (50.32) b [39.36 ;61.90]	**54.32** (50.12) b [43.24 ;65.40]
**Q4**—In general, dengue fever symptoms manifest after an incubation period of *(2–3 days/****4-10 days****/10-14 days/more than 14 days)*	**56.79** (49.64) a [50.52 ;63.06]	**68.35** (46.81) a,c [57.87 ;78.84]	**89.87** (30.36) b [83.07 ;96.67]	**75.31** (43.39) b,c [65.71 ;84.90]
**Q5**—The dengue virus is rampant *(tropical and temperate regions/****tropical regions****/wherever there are bodies of water/it changes every year)*	**28.40** (45.18) a [22.69 ;34.10]	**45.57** (50.12) b [34.34 ;56.80]	**67.09** (47.29) c [56.50 ;77.68]	**66.67** (47.43) c [56.18 ;77.16]
**Q6**—How many dengue serotypes are there? *(1/2/3/****4****)*	**23.05** (42.20) a [17.71 ;28.38]	**44.30** (49.99) b [33.11 ;55.50]	**72.15** (45.11) c [62.05 ;82.26]	**62.96** (48.59) c [52.22 ;73.71]
**Q7**—Which of the following statements is accurate *(answer*: ***dengue fever and malaria are different diseases transmitted by two different species of mosquitoes****)*	**51.85** (50.07) a [45.52 ;58.18]	**64.56** (48.14) a,b [53.77 ;75.34]	**78.48** (41.36) b [69.22 ;87.74]	**71.60** (45.37) b,c [61.57 ;81.64]
**Q8**—The dengue virus is transmitted to humans through bites by mosquitoes of which species? *(Aedes albopictus/****Aedes aegypti****/Anopheles/all species of mosquitos)*	**26.34** (44.14) a [20.76 ;31.91]	**63.29** (48.51) b [52.34 ;74.16]	**43.04** (49.83) c [31.88 ;54.20]	**58.02** (49.66) b,c [47.04 ;69.01]
**Q9**—If a patient obtains a negative result on a rapid diagnostic test (RDT) for malaria, what should be done? *(antimalarial treatments as a precaution/aspirin or ibuprofen/another RDT 48 hours later/****none****)*	**18.52** (38.93) a [13.60 ;23.44]	**29.11** (45.72) a,b [18.87 ;39.35]	**35.44** (48.14) b [24.66 ;46.23]	**45.68** (50.12) b,c [34.60 ;56.76]
**Q10**—Do the different dengue serotypes produce… *(different symptoms but same antibodies/different symptoms and different antibodies/****same symptoms but different antibodies****/same symptoms and same antibodies)*	**35.39** (47.92) a [29.34 ;41.45]	**41.77** (49.63) a,b [30.65 ;52.89]	**55.70** (49.99) b [44.50 ;66.89]	**51.85** (50.28) b [40.73 ;62.97]
**Q11**—After recovering from dengue fever, a patient has lifelong immunity against all serotypes of this disease *(true/****false****)*	**65.84** (47.52) a [59.84 ;71.85]	**64.56** (48.14) a [53.77 ;75.34]	**79.75** (40.45) a [70.69 ;88.81]	**64.20** (48.24) a [53.53 ;74.86]
**Q12**—In recent years, the number of dengue cases in the world has gone down, particularly due to the development of a vaccine *(true/****false****)*	**54.73** (49.88) a [48.43 ;61.04]	**50.63** (50.32) a [39.36 ;61.90]	**56.96** (49.83) a,b [45.80 ;68.12]	**71.60** (45.37) b [61.57 ;81.64]
**Q13**—When a patient is suspected of being infected by dengue fever, a rapid diagnostic test for malaria should still be done *(****true****/false)*	**80.66** (39.58) a [75.66 ;85.66]	**91.14** (28.60) a [84.73 ;97.55]]	**78.48** (41.36) a [69.22 ;87.74]	**81.48** (39.09) a [72.84 ;90.12]
**Q14—**Among the mosquitoes responsible for dengue transmission, both males and females are carriers of the virus *(true/****false****)*	**54.73** (49.88) a [48.43 ;61.04]	**56.96** (49.83) a [45.80 ;68.12]	**67.09** (47.29) a [56.50 ;77.68]	**61.73** (48.91) a [50.91 ;72.54]
Q15* - The mosquito that transmits dengue fever is present mainly in urban areas *(****true****/false)*	**59.33** (49.29) a [51.38 ;67.29]	**65.96** (47.90) a [51.89 ;80.02]	**68.89** (46.82) a [54.82 ;82.95]	**66.00** (47.85) a [52.40 ;79.60]
**Overall results**	**40.60** (13.86) a [38.85 ;42.36]	**52.91** (15.88) b [49.35 ;56.47]	**61.18** (14.78) c [57.87 ;64.49]	**59.50** (17.44) c [55.65 ;63.36]

* Question 15: No video: *n* = 150, Journalistic: *n* = 47, Dramatic: *n* = 45, Animated: *n* = 50

Note. Values within each row that do not share the same letter differ significantly at p<0.05

For the overall score on the 15 questions, a statistically significant difference was seen among the four groups (F(3, 478) = 57.765; *p* <0.001). Tukey’s post hoc tests show that all three videos led to significant rates of knowledge improvement when compared with the comparison group (**40.60%** (95% CI [38.85;42.35]): 12.31% for the journalistic video (**52.91%** (95% CI [49.35;56.47]), 20.58% for the dramatic video (**61.18%** (95% CI [57.87;64.49]), and 18.91% for the animated video (**59.51%** (95% CI [55.65;63.36]). As shown in Table 4, the dramatic and animated videos produced a significantly higher increase in knowledge than did the journalistic video (with respectively 8.27% (*p* = 0.003) and 6.59% (*p* = 0.029) and can be considered equivalent with a difference of 1.68% (*p* = 0.895). In summary, the percentage of correct answers on the knowledge test was significantly higher for participants who viewed the dramatic and animated videos. These results are based on overall score averages and on multiple-choice questions.

For the multiple-choice questions (1 to 10), among participants who viewed the journalistic video there was a statistically significant increase in the percentage of correct responses in five questions out of 10 when compared with the comparison group (Tukey’s post hoc test, *p* <0.05). Among participants who viewed the dramatic and animated videos, nine questions out of 10 obtained higher correct response rates than in the comparison group. Moreover, there was no significant difference between the dramatic and animated videos for all 10 questions (Tukey’s post hoc test, *p* >0.05).

For questions 11 to 15 (with the exception of question 12 (*p* = 0.031), it was difficult to obtain significant results because these were true-or-false questions. In fact, when the mean score for each video was calculated excluding the true-or-false questions, the mean for the comparison group dropped markedly, from 40.60% to 32.65% (Table 5). In contrast, the means for the experimental groups varied by only 1.56% to 3.80%. This is likely due to the fact that the participants had a one-in-two chance of responding correctly.

**Table 5:** Comparison of knowledge acquisition when excluding true-or-false questions.

	No video (n = 243)	Journalistic (n = 79)	Dramatic (n = 79)	Animated (n = 81)
Means **with** true-or-false questions	40.60%	52.91%	61.18%	59.51%
Means **without** true-or-false questions	32.65%	49.11%	59.62%	57.28%

Analysis of each question also revealed a significant knowledge gap. For example, the most important question (Question 9) concerned the treatment to be administered when a patient obtains a negative result on a malaria rapid diagnostic test (RDT). This question was the most missed by the comparison group, with a success rate of only 18.52%.

### Qualitative component

The focus groups and individual interviews showed that video was greatly appreciated as a KT tool. It is easier to take in information from a video than from a written document, as reading is perceived as more tedious.

*“Memory retains visual input more easily*. *Seeing and hearing something makes it more memorable than when it comes via a document*. *When someone gives me a document*, *I look at the title and then I set it aside*.*”* (Student, private school, 2nd year)

This perception was shared by many students. Video as a tool *“breaks the routine”* (instructor, public school). The recreational nature of video was also often mentioned: *“I can’t use my book to enjoy my leisure time*. *But I can do it with video*, *which is also a means of distraction*.*”* (Student, private school, 2nd year). Thematic analysis of the qualitative data revealed that the majority of participants considered videos 2 (dramatic) and 3 (animated) to be the best KT tools. To explain why these two videos also led to better results on the knowledge test, the rest of this section is structured around four themes and certain statistical results are provided to support our analysis.

### 1- Importance of the narrative

#### The importance of storytelling

Participants identified the use of narrative in video 2 (dramatic) as a quality that made this video more attractive than the others. Even though the narrative framework is thin, that is, the story is simply a medical consultation, video 2 (dramatic) tells a story in which there is a progression: an encounter between a doctor and his patient who does not know what she is suffering from and who is surprised by the doctor’s revelations about dengue fever. Participants responses suggest that the use of a story improves information processing and makes the message more persuasive.

*“As a physician*, *you need to know how to examine patients*, *listen to your patients*, *what symptoms they have*, *and then run tests*. *Now*, *what story did the drama present*? *The woman arrived*, *explained her symptoms*, *the physician did the test and saw it wasn’t malaria*. *So he needed to be concerned about dengue*, *because malaria and dengue have the same symptoms*.*”* (Student, public school, 1st year)

The results also reinforce the idea that the absence of an emotional dimension in video 1 (journalistic) handicapped this genre compared to the other two. The objective and emotionless approach of journalism in fact generates a sense of boredom that diverts attention: *“With the journalistic [video]*, *I wondered how long it would last*. *I said to myself*, *I hope it doesn’t go on for an hour*.*”* (Nurse, hospital).

#### The role of role-playing

The results show that the role-playing, which is the backbone of video 2 (dramatic), fosters concentration. This device was recognized as a particularly effective means of transmission because *“it tells the truth”* (student, public school, 3rd year) and because the management of the patient was perceived as being more clearly explained. The actors’ role-playing was considered realistic, which helped with message retention.

*“We’re ‘inside’ the consultation scene*, *which helped me to concentrate*. *Your mind retains it better*. *The day you see a case*, *it takes you back to the image you saw*.*”* (Instructor, public school)

A participant from public school added that the students were able, in this way, to identify with health workers; they felt challenged and involved, which encouraged attentive listening. One nurse (primary health centre) confirmed that this dramatization helped him to identify with the health worker, who became a role model, and to learn how to behave toward the patient. More specifically, it was the alternation between the patient’s questions and the physician’s responses that helped create an engaging story for the viewer. Our results showed that, short of being actively involved, attending a filmed role-playing is equally effective for learning. The effectiveness of the filmed role-playing was largely due to the realism of the scene. For example, according to one public school instructor, the reactions of the patient, who did not contain her astonishment when the doctor insisted on the seriousness of the virus and its mode of transmission, accurately reflected the behaviour of a patient coming for a consultation.

The patient’s reactions in the video are not trivial, as they are helpful for effectively transmitting certain data, even of a more technical nature. Question 6 (*How many different dengue serotypes are known*?) is a good example. The comparison group obtained a very low score (23%), whereas those who viewed the dramatic and animated videos performed well (72% and 63%, respectively). In the animated video, the dengue serotypes were presented in the form of four viruses of different colors. The images were considered to be very clear, and it was not surprising that the question achieved a good response. *“When you watch the animation*, *it’s as if it’s all been laid out for you*. *The information is mapped out and it’s clearer*.*”* (Student, public school, 1st year)

It was more surprising, however, to find that those who had viewed the dramatic video responded even better to this question. In this video, the physician explained the disease to the patient, enumerating the four dengue serotypes by simply counting them on his fingers. The patient was astonished to learn that the four serotypes all produce the same illness, but that the antibodies needed to fight them are different, which means a person can contract four different types of dengue over the course of a lifetime. What emerged from this was that the “explanation–reaction” stratagem in role-playing worked as well as graphic illustrations; this would merit further exploration in a future study.

#### Real-life setting

The journalistic video was the only one filmed in a real primary health centre, a choice of setting that was appreciated by some participants for its realism. In fact, one health worker suggested that the journalistic video could be useful for professionals already on the ground, because it conveyed real life *“in the actual workplace”*. However, even though local images helped the audience identify with the content being presented, this was not enough to hold their attention. As a public school instructor complained, *“The information is presented in the usual way”* and *“there’s nothing new”*. With regard to inattention, question 2, on the breeding grounds of the dengue-carrying mosquito, was revealing. Among the students who viewed the journalistic video, this was the question most missed, with a success rate of only 24.05% (compared to dramatic, 37.97%, and animated, 44.44%). Yet this information was illustrated by several photos of everyday containers (pail, tire, plant pot) that were synchronized with the enumeration in the narration, and which should have made it easier to remember.

#### Varying the rhythm

The structure of video 2 (dramatic), which involved alternating between scenes of consultation and monologues by the physician, with the inherent breaks in rhythm (stop and restart), was perceived as driving the narrative. Participants indicated that the physician’s comments consolidated the knowledge by providing details on what was observed or explained in the consultation scenes.

*“It’s good because he [the physician] sums things up for the audience each time*. *He holds the viewers’ attention… He keeps stopping to address the audience in a serious manner; that’s very important*. *It’s important because we see he’s doing it to capture the viewers’ attention… and he emphasizes certain key points*.*”* (Nurse, primary health centre)

Rather than presenting a continuous narrative, the stratagem of switching between two narrative forms helps maintain the audience’s interest, making it easier for them to understand and assimilate the knowledge.

### 2- Importance of communicators

#### On-screen narrators

In videos 1 (journalistic) and 3 (animated), the narration is off-screen, that is, the narrators are heard but not seen. The conventional and monotone delivery of the journalistic video contributes to the feeling that *“journalism talks about the problem in a general way”* (instructor, public school). The narration (female Burkinabè voice) imitates the objective tone of journalistic reporting and presents the facts without emotion. No point is emphasized; rather, each one follows on the next, with equal importance and without modulation, hence the feeling of a “comprehensive” approach. One public school instructor said that:

*“The journalistic video was not quite sensational enough. We don’t get much of a feeling, we don’t sense the seriousness [of the situation]*. *I didn’t like it… It was less moving.”*

**Fig 4:**
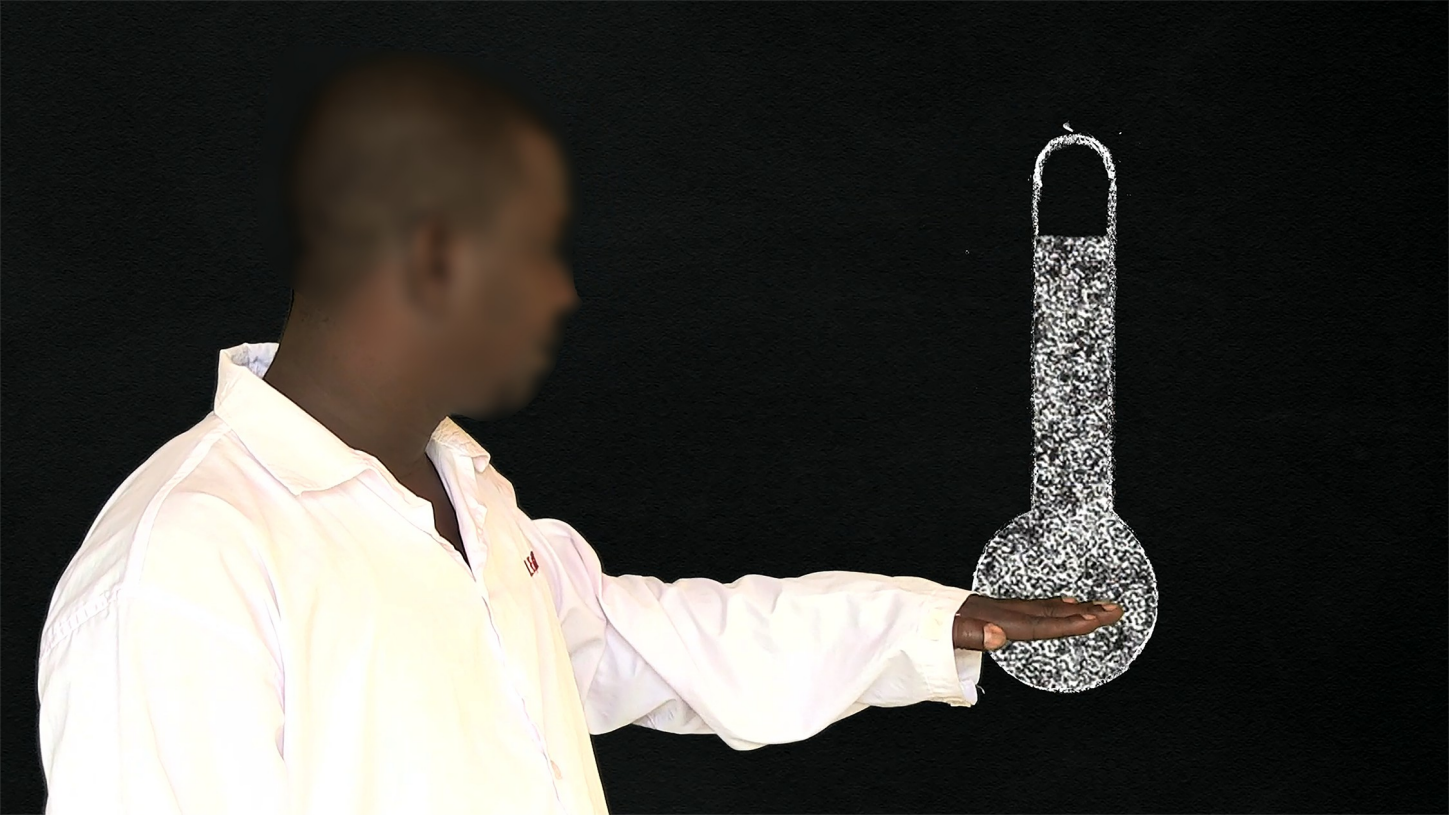
Interaction between the narrator and an infographic image.

In contrast, the narration in video 2 (dramatic), provided by the physician-narrator addressing the audience directly while looking into the camera, appeared to have had a significant impact on message retention. Moreover, the narrator’s role was reinforced by the infographics (Fig 4) that accompanied his monologues. In fact, the physician-narrator character sometimes interacted with short graphic animations embedded behind him. For example, when he was speaking about a lowering of temperature, his arm traced the mercury falling in a thermometer on the screen. *“I think it’s a good thing*, *because he’s explaining what the viewers saw as an image*. *At the same time*, *he’s reinforcing their knowledge at this level*.*”* (Instructor, public school).

#### Working with Burkinabè actors

In the journalistic video, some explanations were given by a nurse, who was quite naturally credible due to his professional status. One might wonder whether having these explanations conveyed by actors would detract from the credibility of the message. On the contrary, said one public school instructor; an actor can make the message clearer, more *“intelligible”*, and more *“credible”*: *“The actor can speak more easily and engage people’s attention*, *which not everyone can do*.*”* Several participants found the nurse interviewed in the journalistic video to be boring. However, using actors to portray the doctor and the patient was considered appropriate by all. Several participants even said the actors could have been more expressive, particularly since patients with dengue fever generally arrive in great pain. *“Most [real patients] lie on the table moaning*. *The woman [actor] didn’t appear sick enough*, *her pain didn’t come across in her physique*.*”* (Nurse, primary health centre). Finally, the vast majority of participants said the Burkinabè nationality of the actors enhanced the credibility of the dramatic video. One public school instructor noted that *“if it’s a black man*, *people will say to themselves that this really is the situation in our country*.*”*

### 3- Importance of visual components

#### Animated images

When addressing the theme of what images are the most appropriate or considered most attractive, the animated video stood out markedly from the other two. One nurse asserted that images were more precise because *“everyone sees and understands the same thing”*. A public school instructor noted that the animated video had *“no images that were not informative and that all the images were focused on information*.*”* Some of the students clearly summarized this perception of the animation video: *“The visual and verbal seem to be better combined*. *In the other videos*, *there’s just the verbal*.*”* (Student, private school, 2nd year).

#### Coordinating verbal and visual

One might wonder whether superposition of the moving image and sound would produce an undesirable duplication effect, but that was not the case. Combining audio and visual gave greater consistency to the message, which was described as “*conveying the maximum information*” (nurse, hospital). The phrase “we are shown”, which recurred in the interviews, revealed that the visual dimension left its mark on people’s minds. This close association between visual and audio freed the viewers from having to make the connection themselves and gave them the impression that the information, thus schematized, was more accessible and easier to remember.

In addition, a visual method that is too general makes the message less clear and can become an obstacle to knowledge transfer. The journalistic video was cited as an example of this. One public school instructor described it as being only a *“briefing on the problem”* and, as such, not geared toward an audience of health professionals. The participants concluded that the journalistic video did not clearly convey information on dengue fever. Yet the narrated text in that video was identical to that of the animated video. The general visual elements of the journalistic video did not sufficiently concretize the information being transmitted verbally, leaving viewers with a nebulous impression of what was actually being communicated.

**Fig 5:**
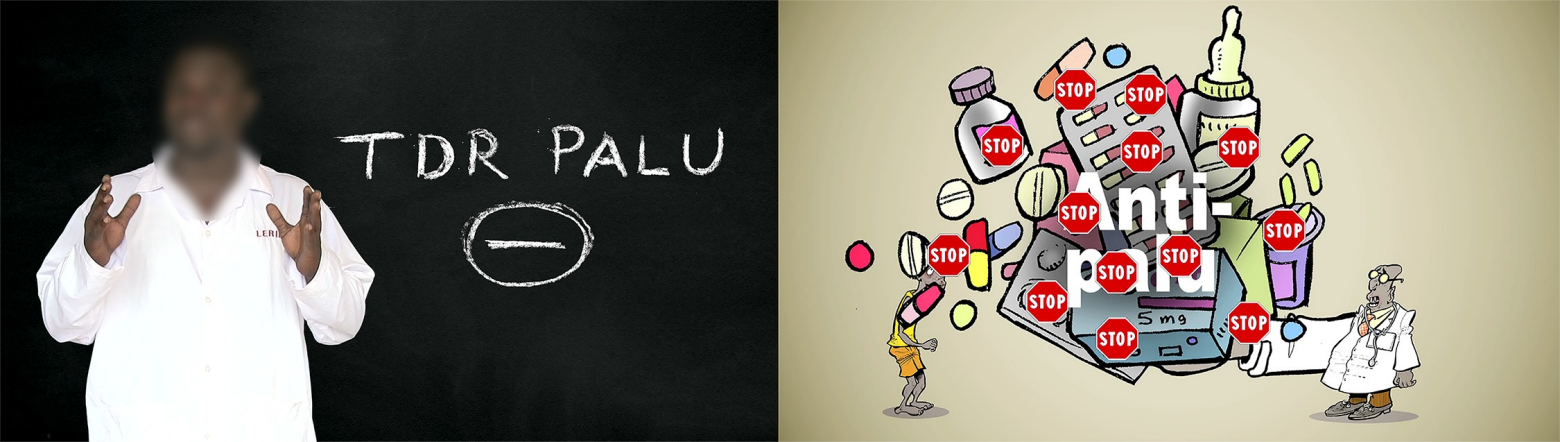
Visual elements of the dramatization and animation videos.

The effectiveness of presenting verbal and visual explanations in a coordinated manner was also revealed by the quantitative results. The question 9 of the knowledge test concerned the recommended management of a patient who obtained a negative result on a malaria RDT. Here again, the journalistic video did not lead to any significant knowledge improvement compared to the comparison group (*p* = 0.241), whereas the improvement was statistically significant for both the animated (*p* <0.001) and dramatic (*p* = 0.015) videos. In both cases, the information was accentuated by striking visuals. In the dramatic video, the physician’s explanations were illustrated by the negative RDT sign as it is generally inscribed by the health worker in the patient’s medical record (Fig 5). In the animated video, the physician holds a mountain of drugs, with “stop” signs appearing on each of them in a burst of sound effects (Fig 5). The low scores obtained by the students who viewed the journalistic video, in which the generic images revealed no details, were very likely due to such lack of attention.

#### Bright colors to capture attention

With respect to the visual elements, the majority of participants expressed a preference for the images of the animated video, identified as the most attractive images, particularly because of the colors:

*“The animation was striking because*, *with the animation*, *we could see the colors better*. *Even if you don’t feel like seeing the colors*, *you’re still drawn to it*.*”* (Student, public school, 3rd year).

One student (private school, 2nd year) noted that the colors helped him integrate the notion of danger: *“In Africa*, *the mosquito is the enemy*. *When we see bleeding*, *the color red*, *we know it means danger*.*”* The red cross that appears on the medications to warn against self-medication was also identified as a danger symbol due to its color.

### 4- Importance of message adaptation

#### Using local language

The results indicated that the tone and the language used by the actors contributed to a better understanding of the message. One nurse (primary health centre) noted that *“the message came across better with the dramatization because the words they used were easier to understand”*. Questioned about understanding the message, a nurse explained:

*“About the way of speaking: if it’s among ourselves, we understand each other*. *But if it’s you, your way of speaking, there are others who don’t easily understand.”*

Both the intonations used by the actors, reflecting the local accent, and their vocabulary were more adapted to the target audience. In this regard, question 1 of the knowledge test was interesting. This question concerned the times of the day when dengue mosquitoes are most likely to bite humans, that is, at dawn and dusk. Students who viewed the dramatic video scored significantly higher (65.82%) on this question than both the comparison group (25.51%) (*p* <0.001), the journalistic video (43.04%) (p = 0.012) and the animated video group (41.98%) (*p* = 0.007). This was also the question on which the students who viewed the animated video obtained the lowest score; it should be noted that the animated video was narrated by a male voice with a European accent. Another possible explanation was that the actor playing the physician in the dramatic video modified the text slightly, replacing the words “dawn” and “dusk”, which were used in the journalistic and animated videos, with “sunrise” and “sunset”.

#### The use of humor

The majority of participants appreciated the humorous tone of the dramatic and animated videos, which held their interest more and, in so doing, enhanced their concentration. One nurse, comparing the animated and journalistic videos, provided a good summary:

*“The animated video is humorously serious*. *We become aware very quickly that the subject is serious even if the language expressing it is not*. *This way*, *we learn more easily and retain it better*. *Using humour increases the chances for maximum retention…*. *The journalistic video*, *very serious*, *requires a strong capacity for attention*, *which can rapidly become distracted*. *Whereas when it’s humorous*, *I’ll concentrate because I’m expecting something interesting to come up next*.*”* (Nurse, hospital).

Here it should be mentioned that the notion that dengue can be fatal came up only in the discussions around the animated video, even though that information was contained in all three videos. This suggests the humor that characterized the animated video did not minimize the seriousness of the message. It was surprising to see, however, that even though all participants agreed that humor facilitates message transmission, their perceptions of what was humorous varied. While some identified the animated video as the most humorous, others preferred the dramatic video. One public school instructor even said he found the tone of the animated video to be *“purely scientific”*. It might therefore have been expected that, within the same context—that of health professionals in Burkina Faso—humor would be decoded in much the same way by all participants. However, the satiric nature of the caricatures presented in the animated video was not grasped by everyone. Some only saw the pictures without noticing their humorous nature. This was surprising, given that the caricatures were drawn by the illustrator of *Journal du jeudi*, a satirical Burkinabè newspaper that was very successful and had become a major societal phenomenon [52].

## Discussion

This study contributes to the advancement of knowledge on what motivates the use of video as a KT tool and practice changes in working contexts where professionals need to know how to respond, quickly and appropriately, to health problems such as dengue and other emerging pathologies in Africa. Given the increasing prevalence of dengue fever and the lack of training among health workers in Burkina Faso [28], there is a need to develop effective KT tools.

The first research question asked whether the videos were an effective KT tool for transmitting research evidence on dengue to health workers in Burkina Faso. Because the groups that viewed the videos learned significantly more than did the comparison group, the present study showed that viewing videos can lead to significant knowledge improvement, as previously shown in the literature [39, 53–55]. The qualitative analysis component of our study showed that health personnel are more inclined to acquire new knowledge by watching a video, rather than reading written documentation, which is considered more laborious, according to them. Video offers an effective way of communicating evidence and facilitates access to such data—a dimension considered essential for ensuring knowledge is applied [7]. The ease with which video can be shared and its playful nature make it a particularly attractive tool for reaching health professionals, given their busy schedules.

Moreover, this study provides an additional contribution to the literature that examines video as a KT tool. Our study argues that the narrative genre of the video influences the interest of participants—a sine qua non condition for the success of a KT exercise. The second research question asked whether participants’ learning varied depending on which video they viewed. In this experiment, results showed that the groups who viewed the dramatic and animated genres increased their knowledge scores significantly more than did the group who viewed the journalistic video. This suggests that the narrative genre of the video influences the viewer’s capacity to retain information, and that some narrative elements foster knowledge transfer more than others.

On that point, the third research question asked what narrative elements (e.g., narration, images, infographics) make a video a more effective KT tool. Therefore, this study identified the importance of four key aspects to consider for a video to be effective: 1) Transmitting information in a narrative form, 2) Choosing good communicators, 3) Creating a visual instrument that reinforces the message and finally, 4) Adapting the message to the local context. As we will see, the different appreciated narrative aspects by the participants are mostly supported by scientific literature. A summary of key elements to consider in producing a video is presented in Table 6.

**Table 6:** Summary of key elements for the ideal video.

**1. HOW?**	-Present knowledge in narrative format (tell a story) -Stage a role-playing scenario (appeal to emotions) -Film the video in a real-life setting (such as a health centre) -Try to vary the rhythm
**2. BY WHOM?**	-Have the narrator speak directly into the camera to address the viewers -Use actors from the target country -Ensure the actors perform energetically and with emphasis
**3. WHAT?**	-Animate cartoon characters (who could reflect the traits of actors on the screen -Coordinate verbal and visual -Use bright colors to capture attention -Use graphic animations that interact with the actors
**4. FOR WHOM?**	-Use the local language with its own expressions and intonations -Have the text reviewed and adapted by health professionals and actors in the target country -Use humour whenever possible

### How? Transmitting information in a narrative form

The narrative is a communication technique in which stories, metaphors, and comparisons are used to enhance the audience’s emotional connection to the information being communicated or to the targeted behaviour [56]. Narrative can be contrasted with factual messages that use statistical evidence, probabilities, and appeals to logic to persuade and motivate people to change their behaviour [57]. Our results actually suggest that viewers’ interest is engaged more effectively when the information in conveyed in a way that helps viewers to identify with the situation being presented and elicits an emotional connection. According to participants, the dramatic video was found to be more effective largely for this reason. A presentation that engages viewers will result in better retention. Also, a recent study shows that a narrative communication delivered through video is more effective than a didactic video to improve attitude and intention to perform a health behavior [32]. Another study shows that viewing a narrative rather than an informational video about a health behavior leads to better information recall several months later [58]. Through the story that is told, people are transported into a situation that amplifies their emotions and influences their attitudes and behaviours [59]. According to Bergsma [60], video enhances the cognitive capacity of memory through the juxtaposition of cognition and emotion, which is often the distinctive feature of animated images and dramatic narratives. More recently, scholars have focused on the importance of storytelling and narrative in policy and politics [61].

The results also showed that the “storytelling” sequences in the dramatic video–dialogue between a physician and a patient—enabled participants to be present where they would not normally be, and as such, were particularly effective [62]. For example, this video allows students to directly observe a medical consultation scene, which would allow better information encoding. While real situations *in situ* are considered to provide the most effective learning environments [63], role-playing is a secondary approach of choice: it is used to reproduce certain critical aspects of the natural environment with the hope that the behaviour reproduced in this alternative framework will accurately reflect the real functioning [64]. Hoeken & Fikkers [65] identified in their study that when people can identify themselves with a character, they would adopt this character's attitudes. In addition, the positive effects of video modeling would be increased “*when the video is racially*, *ethnically*, *linguistically*, *and/or culturally concordant with the target population”* [66]. However, it is important to point out that role-playing cannot fully reflect reality and that a period of retrospection focused on metacognition is needed to ensure the acquired knowledge is transferred [67]. As such, it would be useful to assess how much the learners have actually retained from the role-playing several weeks after the screening.

Another effective narrative aspect of the dramatic video is supported by studies: that is varying the rhythm. Based on a literature review, Berk [68], describes the eight steps required for effective knowledge transfer via video. One of these is to pause the video at any scene to highlight a point. This pause is, in effect, exactly what the physician-narrator accomplishes in video 2, interrupting the scene between himself and his patient to speak to the viewer, either to reinforce the information that has just been presented or to supplement it.

### By whom? Choosing good communicators

Studies [69] previously highlighted the role of narration by suggesting that contiguous presentation of verbal and visual material in videos with integrated narration leads to better learning. Our research suggests the nature of that narration plays a role in information retention: a personalized narration (as opposed to a neutral delivery, as in the journalistic style) delivered by a narrator speaking directly to the viewer *on screen* (as opposed to an off-screen voice) fosters knowledge transfer.

When producing a dramatic video, this study also highlights the importance of mobilizing actors from the target country. This reflects the idea that theatre has the power to give society a representation of itself. In theatre, the public seeks a mirror that will highlight certain aspects of the society to elicit a cognitive or emotional reaction [70]. Theatre is thus a useful tool for constructing a video that can be used to communicate with learners at a deeper level of understanding, by touching their emotions [68, 71]. It also confirms that using local actors and using local languages add to video’s advantages for training purposes [39].

It is also important to ensure that they perform energetically and with emphasis. Regarding the dramatic video, a more dramatic performance by the actors would have amplified the message, in keeping with the notion that, to ensure that spectators grasp the actor’s performance, the latter needs to align his overall expression (body language, facial expression, intonation) with the text [72]. Also, the effectiveness of the dramatic video confirms that the use of actors does not negatively influence the credibility given to the message conveyed in the video.

### What? Creating a visual instrument that reinforces the message

A video is a better KT tool when it includes visual elements—infographics or animations—that illustrate the ideas being presented. These results, confirming that effective understanding of scientific explanations requires mapping between words and images, are consistent with the theory of double coding [73, 74], which posits that information is more easily retained in memory when it can be encoded both pictorially and verbally. This is equally true for video—i.e., moving pictures—because the multimedia auditory/verbal and visual/pictorial stimuli enhance memory and comprehension and enable deeper learning than would any single stimulus on its own [68]. This study actually confirms that, in audiovisual integration and memorization, the details of an image often elude perception unless an accompanying text prompts the viewer’s particular attention [75].

Another important result of this study is the attractiveness of the colors used in the animated video. This was a significative result because it confirms that, for a KT activity to be successful, the information being conveyed needs to be attractive, which requires a good understanding of users’ preferences [7]. This preference for bright colors should be taken into account because it helps with assimilation of the message. Furthermore, color choice is not a trivial matter when attempting to draw people’s attention. In fact, the colors we perceive have not only an aesthetic value, but also a communication value, because of the different associations and meanings they elicit [76, 77]. Like we saw in this study, the color red, which implicitly suggests danger, can be employed effectively to transmit useful information related to a danger [78].

### For whom? Adapting the message to the local context

Video that adopts a humorous tone is considered more captivating than a didactic document and contributes to better retention of content. Yet humor is a fundamentally cultural phenomenon [79]. Some studies have suggested, however, that part of the emotional experience of humor is influenced not only by the context, but also by the internal psychological environment, of which the individual is not always aware [79]. This would, to some extent, explain the divergent perceptions found in our study.

Moreover, the language and intonation used should be adapted to the local context. In this study, word choice was not the only factor that influenced understanding. Certainly, language can influence knowledge transfer in various ways because it represents an essential aspect of culture [80]. Even though a common language (French) was used for the videos, and the narrative text was drafted by a francophone Quebec researcher, the narrators (European and Burkinabè) used a variety of expressive and interpretative devices derived from their respective linguistic systems [81]. It was these expressive devices, the “way of speaking”, which contributed to a better understanding of the material when the narrator was Burkinabè. This affected participants’ ability to assimilate knowledge.

### Limitations

In interpreting the results of the present study, certain limitations should be kept in mind. First, even though the sample can be considered sufficient, it is nevertheless a small sample. Also, as the conditions for video projection differed from one class to another, it would be advisable to set up a projection device to avoid such irregularities. Along the same lines, it would have been preferable not to have the experimental groups interacting with each other outside the classroom between projections (Fig 3), to avoid the risk of contamination. Still, the students appeared to have respected the instruction not to share information about the videos.

The measurement instrument developed for this study—that is, the knowledge test—could be improved. It would have been preferable to validate it beforehand with Burkinabè nurses and instructors. It could also be made more precise by replacing the true-or-false questions with multiple-choice questions. We could also have measured other socio-demographic variables in the questionnaire such as having already been diagnosed with dengue fever or not. Moreover, a right-after-training knowledge test was used in this research to evaluate the effectiveness of video as a KT tool. It would be helpful if future studies could measure actual changes in behavior that occur during medical consultation. It is quite possible that participants may forget some knowledge about the diagnosis of dengue in the absence of appropriate reinforcement because transferring learning to behavior is an important issue in professional training. To improve the sustainability of our initiative, a modified version of the video used in this study is available online and can be downloaded so that the professionals can access it at any times [82].

The focus groups and individual interviews were led by the first author of this article. This may have introduced a situational bias among participants and influenced their perceptions of the usefulness of video as a KT tool. Moreover, the virulence of the dengue fever epidemic that had erupted several months before the study and the threat of a new epidemic may have influenced participants’ motivation and enthusiasm for the video tool. Also, given that the first author of this article has worked in the field of documentary cinema, her attraction to video may have influenced her interpretation of the qualitative results.

Lastly, the quantitative data were obtained from students, whereas the video is intended for nurses in the professional setting, and it is possible that there is a gap between these two populations. That being said, however, the impressions that emerged in the individual interviews with health professionals overlapped on several points with those of the student focus groups. This suggests that the results regarding the strengths and weaknesses of each video might be generalizable to health professionals. Finally, the extent to which the results of this study in Burkina Faso would be applicable to other countries is uncertain, given that cultural context plays a large role in how the videos are received and perceived. Despite the above-mentioned limitations, every effort was made throughout the study to ensure scientific rigour.

## Conclusion

This innovative study concludes that a video's narrative genre significantly influences the subsequent knowledge acquisition. This study also identified the narrative elements that allow a video to be as effective as possible according to Burkinabè healthcare providers. We therefore recommend that more video-based tools should be developed to transfer knowledge to health professionals. New videos could be created to fill certain knowledge gaps or even simply to update knowledge. Further studies should be conducted to better understand what knowledge is actually retained and applied over the longer term, and whether the interest generated for this type of training can be sustained. This would make it possible to develop KT video tools that are better adapted to the needs of health workers and to ensure their wide dissemination. Especially in the context of constant circulation of new diseases, such as COVID-19, it is even more important to support and train public health professionals because they need to know how to respond, quickly and appropriately. In conclusion, if you consider using video as a KT tool, we also recommend having recourse to video professionals in order to ensure their quality and effectiveness.

## Supporting information

S1 Appendix Questionnaire—knowledge test on dengue. (DOCX)

S2 Appendix Discussion grid. (DOCX)
