# Supplementary material for: Video as a public health knowledge transfer tool in Burkina Faso: A mixed evaluation comparing three narrative genres
Source: PLoS Negl Trop Dis. 2020 Jun 10;14(6):e0008305. doi: 10.1371/journal.pntd.0008305 (PMC7286479; doi:10.1371/journal.pntd.0008305)
Supplement: S1 Appendix — (DOCX) [file pntd.0008305.s001.docx]

## Appendix 1 – Knowledge test

**I.** **Please fill in the oval corresponding to the version of the questionnaire you were given:**

a) Version 1 b) Version 2

**II.** **You are**:

1. Female b) Male

**III. What is your age?**

1. 21 to 25 years b) 26 to 30 years c) 31 to 35 years d) more than 35 years

**IV. How many years of experience do you have in urban settings?**

a) 0 b) 1–2 c) 3–4 d) 5–6 e) more than 6

**V. How many years of experience do you have in rural settings?**

a) 0 b) 1–2 c) 3–4 d) 5–6 e) more than 6

**VI. What is the last diploma you received?**

a) Primary level, CEP
b) Secondary level 1,  BEPC (CAP, BEP)
c) Secondary level 2,  Baccalaureate
d) DEUG / Licence

e) Master’s 1 / Master’s 2 (DESS-DEA)

**1. The risk of being infected with dengue fever is highest:**

a) At night.

b) At sunrise.

c) At sunset.

d) b and c.

**2. The mosquito responsible for dengue fever transmission lays its eggs:**

a) In rain puddles.

b) In ponds.

c) In everyday containers that hold water.

d) In large bodies of water, such as dam reservoirs.

e) All of the above.

**3. Every year, globally, the number of deaths due to dengue fever is estimated to be:**

a) 10,000

b) 20,000

c) 30,000

d) More than 30,000

**4. In general, dengue fever symptoms manifest after an incubation period of:**

a) 2 to 3 days

b) 4 to 10 days

c) 10 to 14 days

d) More than 14 days

**5. The dengue virus is rampant:**

a) In both tropical and temperate regions.

b) In tropical regions.

c) Wherever there are bodies of water.

d) Impossible to answer, the virus changes location every year.

**6. How many dengue serotypes are there?**

a) 1

b) 2

c) 3

d) 4

**7. Which of the following statements is accurate:**

a) Dengue fever and malaria are two names for the same disease.

b) Dengue fever is a severe form of malaria, commonly referred to as “palu-dingue”.

c) Dengue fever and malaria are different diseases transmitted by two different species of mosquitoes.

d) Dengue fever and malaria are different diseases transmitted by the same species of mosquitoes.

**8. The dengue virus is transmitted to humans through bites by mosquitoes of which species?**

a) Aedes albopictus.

b) Aedes aegypti.

c) Anopheles.

d) All species of mosquitoes can be carriers.

**9. If a patient obtains a negative result on a rapid diagnostic test (RDT) for malaria, what should be done?**

a) Prescribe antimalarial treatments as a precaution.

b) Prescribe aspirin or ibuprofen.

c) Recommend doing another RDT 48 hours later.

d) None of the above.

**10. Do the different dengue serotypes:**

a) Produce different symptoms, but the antibodies to fight them are the same.

b) Produce different symptoms, and the antibodies to fight them are different.

c) Produce the same symptoms, but the antibodies to fight them are different.

d) Produce the same symptoms, and the antibodies to fight them are the same.

**TRUE OR FALSE**

**11. After recovering from dengue fever, a patient has lifelong immunity against all serotypes of this disease.**

a) True

b) False

**12. In recent years, the number of dengue cases in the world has gone down, particularly due to the development of a vaccine.**

a) True

b) False

**13. When a patient is suspected of being infected by dengue fever, a rapid diagnostic test for malaria should still be done.**

a) True

b) False

**14. Among the mosquitoes responsible for dengue transmission, both males and females are carriers of the virus.**

a) True

b) False

**15. The mosquito that transmits dengue fever is present mainly in urban areas.**

a) True

b) False
