# Supplementary material for: Video as a public health knowledge transfer tool in Burkina Faso: A mixed evaluation comparing three narrative genres
Source: PLoS Negl Trop Dis. 2020 Jun 10;14(6):e0008305. doi: 10.1371/journal.pntd.0008305 (PMC7286479; doi:10.1371/journal.pntd.0008305)
Supplement: S2 Appendix — (DOCX) [file pntd.0008305.s002.docx]

## Appendix 2

##

**Discussion grid**

**Comparison of three videos on dengue fever as a knowledge transfer tool**

**Description of the project**

This interview is part of a large project being conducted by a team of researchers from the University of Montreal and the Health Equity program. The purpose of this meeting is for us to learn about your perceptions following the screening of three videos on dengue fever designed by the research team. **The main objective of this discussion is to understand which of the three videos seems to you to be the best tool for knowledge transfer**. Your opinion is of great importance in understanding how we could improve this knowledge transfer tool.

All information provided will be kept **strictly confidential**, and your answers will be sent only to the team’s principal investigator (Catherine Hébert). Also, any information provided will be treated and presented **anonymously** so that it is not possible to identify you.

**Voluntary participation and right to withdraw**

You are free to accept or refuse to participate in this research project. You may withdraw from this study at any time, without giving a reason. You have only to notify the contact person of the research team by simple verbal notice.

**Introduction**

“During this discussion, I will ask you various questions to better understand the value of each video as a knowledge transfer tool.

Do you have any **questions** before we begin the discussion?”

**Discussion**

1. Which of the three videos did you prefer?

2. What elements of this video lead you select this one rather than the other two?

3. In your view, which video is the best tool for transmitting knowledge to health professionals? What makes it more effective than the others?

4. Which of the three narrative styles did you prefer? What makes it better?

5. From a visual perspective, which of the three videos do you find most attractive? Which one would you be likely to watch to the end if you received it on your mobile phone?

6. Did you find that one or more of the videos adopted a humorous tone? What impact can humour have on the reception and retention of the knowledge transmitted in the video?

7. If you want to download a video, what is the maximum number of megabytes that you consider acceptable?

8. What did you think of the length of these videos?

9. Do you have any further observations or feedback you would like to share?

# 
